# Chronic Hepatitis C in Saudi Arabia: Three Years Local Experience in a University Hospital

**DOI:** 10.5812/hepatmon.6178

**Published:** 2012-09-30

**Authors:** Hisham O Akbar, Ahmad Al Ghamdi, Faten Qattan, Hind I Fallatah, Maha Al Rumani

**Affiliations:** 1 Department of Internal Medicine, King Abdul Aziz University Hospital, Jeddah, Saudi Arabia; 2 Molecular Biology Department, King Abdul Aziz University Hospital, Jeddah, Saudi Arabia

**Keywords:** Hepatitis C, Chronic, Saudi Arabia, Liver Cirrhosis, Genotyping Techniques, Carcinoma, Hepatocellular, Ribavirin

## Abstract

**Background:**

Chronic hepatitis C (CHC) is a global infection. In Saudi Arabia, the prevalence of CHC is declining due to the implementation of a blood screening program. However, CHC still remains a leading cause of liver cirrhosis and hepatocellular carcinoma.

**Objectives:**

This is a retrospective study of CHC patients at the King Abdul Aziz University Hospital, Jeddah, Saudi Arabia.

**Patients and Methods:**

Out of a total of 291 CHC patients from the hepatology clinic at King Abdul Aziz University hospital, Jeddah, 279 patients were included in the present study. They were primarily male (152, 54.5%), with a mean age of 50.41 ± 1.72 years. The majority of patients were either Saudi (108, 38.7%) or Egyptian (60, 21.5%). A total of 61 patients received combination treatment with pegylated interferon and ribavirin, and one patient with sickle-cell anemia received pegylated INF monotherapy. Demographic, clinical and laboratory features of the CHC patients, and their responses to treatment were studied.

**Results:**

Decompensated cirrhosis was documented in 60 patients (21.5%), and hepatocellular carcinoma in 14 (5%). The mean level of serum alanine aminotransferase was 83.6 ± 231 u/L. The predominant genotype among the 70 patients tested, was genotype 4, followed by genotype 1 (39 and 18 patients, respectively). The sustained viral response (SVR) rate was 82.99%. The main predictive factors for SVR were baseline HCV viral load and rapid virologic response (RVR). The mean duration of follow-up was 4.2 ± .85 years. There were 24 patients who had liver disease-related mortality.

**Conclusions:**

our data showed that 22% of CHC patients progress to cirrhosis and another 22% had treatment. Liver related mortality was more common in patients with advanced cirrhosis.

## 1. Background

Chronic hepatitis C (CHC) is a global problem with a variable prevalence in different countries (1). It is estimated that 140-170 million individuals are chronically infected with the hepatitis C virus (HCV), and 3-4 million individuals are infected annually (2). HCV is a leading cause of end-stage liver disease and hepatocellular carcinoma (HCC) (3). HCV transmission is usually an outcome of blood-borne infection, following the transfusion of infected blood products or the sharing of needles with HCV-infected individuals (eg, intravenous drug users), many other patients were reported to have developed the infection during hemodialysis treatment (3, 4). HCV infections in the Kingdom of Saudi Arabia (KSA) were usually acquired as an outcome of the transfusion of infected blood products, before the implementation of blood donation screening programs (5). However, the prevalence of CHC in the KSA has been diminishing steadily over the last decade as a result of these programs (5, 6). Furthermore, the use of polymerase chain reaction (PCR) techniques for screening blood donors is expected to enhance the reduction in the rate of HCV transmission in the future (7, 8). Acute HCV infection is mostly asymptomatic, but the majority (up to 80%) of these patients will progress to chronic liver disease (CLD) (7-9).

There are six genotypes of HCV (1-6) with variable distributions in different regions of the world (7, 8, 10). The predominant genotype in the KSA is genotype 4, followed by genotype 1 (5, 11). These two genotypes are more difficult to treat, compared to genotypes 2 and 3 (8). Genotypes 2 and 3 are recognized less frequently among CHC patients from the KSA (5, 11). The current standard treatment for CHC is the combination of pegylated interferon (PEG-INF) and ribavirin for 48 weeks for genotypes 1 and 4, and 24 weeks for genotypes 2 and 3 (7, 8, 12-14). This treatment is effective in up to 70-95% of patients with genotypes 2 and 3, 44-54% of patients with genotype 1, and 58-86% of patients with genotype 4 (7, 8, 12, 14-17). Treatment response is determined by the sustained virologic response (SVR), which is defined as a negative HCV-PCR result, six months after the end of treatment (7, 8, 14, 15). The SVR is predicted by multiple factors other than the HCV, including; the patient’s gender, viral load and rapid virologic response (RVR), which is defined as either a negative HCV-PCR result after four weeks of treatment or a 2 log reduction in HCV-PCR from the baseline value after four weeks of therapy (7, 8, 16, 18). Alternative treatment options are available for those patients who exhibit a partial response (defined as a greater than 2 log drop in HCV-PCR at week 12, but with detectable HCV-RNA at weeks 12 and 24), null response, or a non-response defined respectively as (less than 2 log decrease in HCV RNA level from baseline at 12 weeks of therapy, and failure to clear HCV RNA from serum after 24 weeks of therapy) (7, 8, 14, 19). Alternative treatment options include; retreatment with combination therapy, extended treatment for 72 weeks or weight-based ribavirin treatment (7, 8, 19, 20). The addition of new, direct-acting antiviral (DAA) medications, such as boceprevir and telaprevir, to the standard therapy is another alternative for those who did not respond to a previous PEG-INF and ribavirin combination. This approach can also be used as the initial treatment strategy in individuals with genotypes that are known to be difficult to treat (7, 8, 21, 22). Untreated CHC patients, and those who do not respond to treatment, carry the risk of progression to decompensated cirrhosis and hepatocellular carcinoma (8, 23, 24).

## 2. Objectives

To study the clinical, laboratory features and follow-up outcome of both treated and untreated CHC patients at the hepatology clinic from King Abdul Aziz university hospital Jeddah.This was a three year descriptive study on the clinical outcomes and treatment responses among CHC patients at King Abdul Aziz University Hospital Jeddah.

## 3. Patients and Methods

This was a retrospective descriptive study designed to investigate the clinical and laboratory features, as well as the treatment responses, among CHC patients at the Hepatology Clinic at the King Abdul Aziz University Hospital, Jeddah, Saudi Arabia, from January 2007 to December 2009. The study group included all patients who were diagnosed with CHC during the study period, using both an enzyme-linked immunosorbent assay (ELISA) to assess hepatitis C virus antibodies and PCR to assess HCV ribonucleic acid (HCV-RNA). Patients were excluded if the patient records were incomplete, a loss to follow-up, or other reasons and if they had a coinfection with HIV or HBV. A total of 291 patients tested positive for HCV-PCR during the study period. The final analysis included 279 patients and the causes for exclusion were; incomplete data for four patients, HBV coinfection in five patients and HIV coinfection in 3 patients. Most of the patients (152, 54.5%) were male (the demographic data are presented in (Table 1). The mean patient age was 50.41 ± 1.72 years, and there was no difference in mean age between the male and female patients. The majority of the patients were Saudi (108, 38.7%) and Egyptian (60, 21.5%) (Table 1).

**Table 1:** Patient Demographic Data and Serum Alanine Aminotransferase Levels

	**Nationality**			**Age, y**	**ALT, U/L**
****	**Saudi, No.**	**Egyptian, No.**	**Other, No.**	****	****
**Gender, Mean ± SD**				50.41 ± 1.72	83.6 ± 23.1
Male	56	42	54	50.26	98.6
Female	25	18	57	50.57	65.6
**Total, No.** (%)	108 (38.7%)	60 (21.5%)	111 (39.8%)	-	-

Abbreviation: ALT, alanine aminotransferase.

For each patient, we collected demographic data including; age, sex and nationality. We also obtained clinical data including; whether the patient had a compensated or decompensated disease (defined as the presence of ascites, hepatic encephalopathy, variceal bleeding and thrombocytopenia, ie, platelet count of less than 100/ml due to hypersplenism from portal hypertension). We also looked for the presence of autoimmune disease or inherited hemolytic disorders, such as sickle-cell anemia (SCA), and thalassemia. For patients with chronic renal failure, their history of hemodialysis was also included. Normal results of a complete blood count (CBC) were defined as follows; white blood cells (WBCs) (3-11 k/ul), hemoglobin (Hg) (12-17 g/dl), and platelet count (PLC) (100-400 k/uL). Liver function tests were assessed using the Dimension clinical chemistry system based on the use of the Flex reagent cartridge. (Dade Lhrlnq Inc. Newark. DE 19714. U S A) Normal results from these tests were defined as follows; serum alanine aminotransferase (ALT) (30-65 U/L), aspartate aminotransferase (AST) (15-37 U/L), alkaline phosphatase (ALP) (50-136 U/L), gamma-glutamyl transferase (GGT) (5-85 U/L), total protein (TP) (64-82 g/L), albumin (Alb) 35-(50 g/L), total bilirubin (0-17 µmol/L), and direct bilirubin (0-5 µmol/L). The ALT level was also measured every four to six months during the follow-up period, if the patient received treatment, the ALT result was also obtained at three to six months from the start of the treatment and after stopping treatment. The level of HCV-RNA was measured by PCR using a COBAS AMPLICOR Analyzer after automated nucleic acid extraction with Cobas AmpliPrep (Roche dignostic, Rotkreuz, Switzerland). This was performed at the time of diagnosis, and then subsequently every three to six months. HCV genotypes were determined using the same PCR method. If the patient had undergone treatment, HCV-RNA was also measured four weeks after the start of treatment by the rapid virologic response (RVR) if available, at 12 weeks as the early virologic response (EVR), at 24 weeks as the end-of-treatment response (ETR) for genotypes 2 and 3 and as follow-up for genotypes 1 and 4, and at 48 weeks (ETR) for genotypes 1 and 4. HCV-PCR for SVR was performed at six months after the termination of treatment in those who had completed the treatment. The results of the abdominal ultrasound and/or the CT scan performed during the study period were also included in the analysis to look for radiological evidence of; portal hypertension, ascites and focal liver lesions. For all treated patients, we collected data on; treatment duration (complete or incomplete), treatment side effects, treatment response, treatment failure and recurrence of the virus after ETR. We also reported the clinical outcomes of the patients as observed at the end of the study period. The existence of HCC was also noted. We used the Statistical Package for Social Science (SPSS 16 Chicago USA) to obtain the means, standard deviations and frequencies. An independent t-test was used to compare the means, and a chi-square test was used to analyze non-numerical values.

## 4. Results

A total of 60 patients (21.5%) displayed clinical and radiological evidence of decompensated cirrhosis, either at presentation or during the follow-up period. During the follow-up period, we observed HCC in 14 patients. Most of the patients (11) who had HCC were male, and three were female. These patients were older compared to the non-HCC patients (means, 64.36 and 49.60 years, respectively; P = .037). Abdominal ultrasound showed evidence of fatty liver in 47 non-cirrhotic patients. Among those 47 patients, six had high levels of serum lipids. Only 70 patients were tested for the HCV genotype, and the majority were identified as genotype 4 (Table 2). Twelve patients had an associated autoimmune disease, and all except one were female. Eleven patients had a history of sickle-cell anemia (SCA), or thalassemia major with a history of frequent transfusions (Table 3). Hemodialysis-acquired infections were reported in six patients (2.2%). The mean serum ALT for all patients was 83.6 ± 23.1 IU/L. There was no difference in the mean ALT levels between the male and female patients or between compensated and decompensated patients, but there was a significant difference among the different genotypes as determined by an analysis of variance (ANOVA) (F = 2.78 and P = 0.034). The mean serum albumin level was 31.28g /l and the mean serum bilirubin level was 23.33 µmol/L (Table 4). Male patients had significantly higher HCV-RNA values compared to females (1.29x 106 and 8.3112 x 105 IU/ml respectively; P = 0.015). Similarly, non-cirrhotic patients had higher HCV-RNA values compared to patients with decompensated cirrhosis (1.283 x 106 and 5.7776 x 105 IU/ml, respectively; P = 0.033). There was no difference in HCV-PCR values among HCV genotypes. Combined treatment with PEG-INF and ribavirin was administered to 61 patients. One patient with SCA and CHC received PEG-INF mono-therapy. The treatment was completed in 47 patients. In 10 patients, the treatment was stopped as a result of either side effects or a lack of response. Five patients were still receiving treatment during the analysis of these data. One patient with genotype 1 and one with genotype 4 stopped the treatment at 30 weeks, but they both achieved SVR. The number of treated females was greater than the number of treated males, 32 (51.6%) and 30 (48.4%), respectively. These patients were primarily Saudi (24, 38%) or Egyptian (19, 30.6%). Treatment side effects were reported in 31 patients; the most frequent side effects in the majority of patients were fatigue and neutropenia, which were improved in all patients following a PEG-INF dose adjustment (Table 5 for a list of treatment side effects). The mean serum ALT level in the treated patients was 82.5 U/L. The majority of the treated patients had genotype 4 (30 patients), followed by genotype 1 (20 patients). SVR was achieved in 39 out of the 47 (82.99%) patients who completed the treatment. SVR was not achieved in eight patients; three had genotype 1, three had genotype 4 and two had an unknown genotype, but both completed the treatment for 48 weeks. Patients who achieved SVR had a lower baseline HCV-RNA level compared to those who did not achieve SVR (9.500 x 105 and 3.315 x 106 IU/L, respectively; P = 0.012). Similarly, patients who achieved RVR had HCV-PCR values that were lower compared to those who did not achieve RVR (1.432 x 106 and 3.258 x 106 IU/ml, respectively = 0.04). Patients who had RVR also achieved higher rates of SVR. Other factors such as; age, sex, nationality and serum ALT, did not have a significant impact on the SVR (Table 6). We also found post-ETR recurrence of CHC in four patients; one had genotype 1, two had genotype 4 and one had genotype 3 with a very high baseline viral load. In the treated patients, the serum ALT at 24 weeks of treatment was lower compared to the pretreatment levels (44.9 and 83.4 U/L, respectively; P = < 0.001). Similarly, patients who achieved SVR had lower levels of serum ALT at 24 weeks compared to non-responders (40.6 and 53.25 Ul/L, respectively; P = 0.01). Our data showed that patients who had decompensated cirrhosis had greater liver disease-related mortality rates compared to compensated patients (24 vs. two patients, respectively; P < 0.001). Another 25 decompensated patients were lost to follow-up and 11 were still alive at the end of the study. The mean duration of follow-up for all patients was 4.2 ± 0.85 years.

**Table 2:** Hepatitis C Virus Genotype According to Sex, for the 70 Patients With Hepatitis C Virus Genotyping

	** Hepatitis C Virus Genotype, No.**					
	**1**	**2**	**3**	**4**	**Unknown**	**Total**
**Male**	5	4	2	22	1	34
**Female**	13	3	3	17	0	36
** Total**	18	7	5	39	1	70

**Table 3:** Number of Patients With a Chronic Hepatitis C-Associated Autoimmune Disease or Inherited Hemolytic Disorder in Relation to Sex

****	** Autoimmune Disease, No.**				**Sickle-Cell Anemia or Thalassemia, No.**
	**Hypothyroidism**	**Thyrotoxicosis**	**SLE**	**RA**	
**Male**	0	1	0	0	6
**Female**	4	1	3	3	5
**Total**	4	2	3	3	11

Abbreviations: SLE, systemic lupus erythematosus; RA, rheumatoid arthritis.

**Table 4:** Mean Laboratory Results for All Patients

	**Mean ± SD**
**Albumin, 34-50 g/L**	31.34 ± 1
**Bilirubin, µmol/L**	23.33 ± 6.8
**ALP, 50-136 U/L**	108.4 ± 6.6
**GGT, 5-85 U/l**	125.5 ± 0.25
**Total protein, 64-88 g/L**	73.6 ± 1.3
**Hg, 12-15 g/dl**	12.2 ± 0.4
**Platelets, 150-450 K/µl**	202.7 ± 13
**WBC, 4.5-11.5 K/µl**	13.24 ± 9

Abbreviations: ALP, alkaline phosphatase; GGT, gamma-glutamyl transferase; Hg, hemoglobin, WBC, white blood cells.

**Table 5:** Treatment Side Effects

**Side Effect**	**Patients No.**
**Leukopenia**	22
**Hypothyroidism and low white blood cell count**	4
**Severe anemia**	1
**Flare-up of autoimmune disease**	1
**Thyrotoxicosis**	1
**Low platelet count**	1

**Table 6:** Linear Regression Analysis for Factors Predicting Sustained Virologic Response

****	**Standardized Beta Coef-ficients**	**t**	***P*value**
**Age (21-66),y**	0.233	1.282	0.214
**Sex (17 male and 19 ** **Females had SVR)**	-0.335	-1.911	0.070
**HCV genotype**	-0.064	-0.388	0.702
**RVR **	0.459	2.604	0.017
**ALT at diagnosis**	-0.293	-1.623	0.214

Abbreviations: ALT, alanine aminotransferase; HCV, hepatitis C virus; RVR, rapid virologic response.

## 5. Discussion

Our study showed that CHC is frequently diagnosed among liver disease patients at the King Abdul Aziz University Hospital, Jeddah. We observed that 291 patients were newly diagnosed over the course of three years. The majority of patients in our cohort were males in their 50s. These findings are consistent with previous data on CHC from other Saudi Arabian researches (5, 25, 26). In our study, the number of Saudi patients was greater compared to other nationalities. The Madani report in collaboration with the Ministry of Health showed a similar finding (26). However, Mimish et al. reported a lower incidence of CHC among Saudi patients compared to non-Saudis (25). The differences in these data are most likely due to variations in the populations of the different regions of the country that were studied. Madani published all of the cases of HCV infection reported to the Ministry of Health, from all regions of the country, over the course of eleven years (26). In a study of HCV from the KSA, Shobokshi et al. reported similar findings (27). The predominant genotype in our cohort was genotype 4, followed by genotype 1. This finding is consistent with those in other reports on HVC genotyping from Saudi Arabia (5, 27, 28). One-fifth of the patients had decompensated cirrhosis at diagnosis or during the follow-up period. This percentage is similar to other recently reported international data (29, 30). Kanwal et al. showed in their study that the rate of progression to cirrhosis among CHC patients had increased from 9.1% in 1996 to 19% in 2006. HCV is a leading cause of HCC in the KSA (31). In our cohort, we reported HCC at a rate close to 5% (14/279), which is higher than the value recently reported by Kanwal et al. (31). A total of 70 patients underwent HCV-PCR testing, almost all of whom had either received or planned to receive treatment. However, this finding indicates that only about one-quarter of the CHC patients in this study had either received treatment, or had been considered for treatment. This figure is equivalent to international reports on treated CHC patients (32). The cost-effectiveness of conducting HCV-PCR testing for CHC patients who are not candidates for treatment, may need to be evaluated in future studies. Twelve of the patients in our cohort had AIDs. HCV is thought to trigger the immune system and is known to be associated with certain autoimmune phenomena such as livedo reticularis (33). Transfusion-related CHC in patients with SCA and thalassemia was associated with blood transfusions which had occurred before the start of the blood donor screening programs. The development of these programs and, in particular, the use of the PCR method for the detection of viral infections among blood donors, reduced the transmission rate of HCV (5, 6, 34). HCV transmission among hemodialysis patients is of great concern in some hemodialysis units (35, 36). In our study, 2.2% of patients had dialysis-related HCV transmission. Serum ALT levels were similar in males and females, which may indicate that the normal difference in serum ALT levels between males and females is lost in CHC patients (37). In our cohort, HCV-PCR levels were higher in male, compared to female patients. This finding is consistent with previously published data (38). The level of HCV-PCR was not different in patients with decompensated, compared to compensated cirrhosis, and the HCV-PCR level was not different in patients with HCC compared to non-HCC patients (38). A total of 61 patients were treated with standard combination therapy, and they achieved an SVR rate comparable to both local and international rates (7, 8, 16, 39, 40). On the other hand, the percentage of untreated patients in our cohort is also similar to international figures, in that the majority of CHC-infected patients are not treated and will ultimately progress to decompensated cirrhosis and its associated complications (7, 8, 29, 40). The use of combination therapy in patients with hemoglobinopathies is less effective, because of secondary iron overload, and ribavirin cannot be used due to the risk of increased hemolysis (7, 8, 18). In our study, the one patient who received pegylated INF monotherapy had an ETR, but not a SVR. The determining factors for SVR in our cohort, were the baseline HVC-PCR value and the RVR; this finding is similar to previously reported data (7, 8, 16). Four patients, who had HCV recurrence after ETR, had very high baseline HCV-PCR values. Extended treatment with combination therapy was effective in achieving SVR in our patients who had a recurrent genotype 1 HCV. This regimen is acceptable in difficult-to-treat CHC patients (7, 8, 41). The side effects of the treatment with a combination of pegylated INF and ribavirin in our patients were similar to those described previously (7, 8), but none of the patients in this study required a reduction in the dose of ribavirin to less than 800 mg daily. In Conclusion: our study showed that CHC in Saudi Arabia is similar to some international figures, in its clinical outcomes and treatment response, but on the other hand it was better in the treatment response than other data on genotype 1. Similarly most CHC patients in our cohort were not treated. Future prospective similar multicenter studies from Saudi Arabia will be helpful in more understanding of the disease in the country and will involve more patients in the treatment plans. On the other hand other studies that will investigate the uses of new treatment regiments for CHC like DAA are a future goal for non-responders. Another future target is to study of the local genetic factors that will predict treatment response and will tailors treatment plans.
